# Synthetic free fatty acid receptor (FFAR) 2 agonist 4-CMTB and FFAR4 agonist GSK13764 inhibit colon cancer cell growth and migration and regulate FFARs expression in in vitro and in vivo models of colorectal cancer

**DOI:** 10.1007/s43440-024-00667-5

**Published:** 2024-10-21

**Authors:** Agata Binienda, Katarzyna Owczarek, Maciej Sałaga, Jakub Fichna

**Affiliations:** Department of Biochemistry, Faculty of Medicine, Medical University of Lodz, Mazowiecka 5, Łódź, 92-215 Poland

**Keywords:** AOM/DSS-induced colitis-associated colorectal cancer, Cancer cell viability, Cell migration, And invasion assays, Free fatty acid receptor type 2, Free fatty acid receptor type 4

## Abstract

**Introduction:**

Free fatty acid receptors (FFARs) are G protein-coupled receptors that divide into 4 subtypes; FFAR2 and FFAR3 are activated by short-chain fatty acids, while FFAR1 and FFAR4 - by long-chain fatty acids. Recent studies show the potential involvement of FFARs in the pathophysiology of colorectal cancer (CRC). A decrease in *FFAR2* and *FFAR4* gene expression is observed in patients with CRC. The aim of our study was to evaluate the anti-cancer effect of FFAR2 and FFAR4 stimulation by selective synthetic agonists in in vitro and in vivo models of CRC.

**Materials and methods:**

FFAR2 agonist, 4-CMTB, and FFAR4 agonist, GSK137647 were used. Cell viability (CCD 841 CoN and SW-480) was determined after 48 h incubation with tested compounds using MTT assay. Real-time qPCR and Western Blot were used to identify changes in FFARs expression. Migration and invasion were characterized by commercially available tests. Colitis-associated CRC (CACRC) mouse model was induced by azoxymethane and dextran sodium sulfate.

**Results:**

4-CMTB and GSK137647 significantly reduced cancer cell growth as well as migration and invasion capacities. Both synthetic compounds increased *FFAR2* and *FFAR4* expression in SW-480 cells. Neither 4-CMTB nor GSK137647 influenced the course of AOM/DSS-induced CACRC in mice, however, 4-CMTB elevated FFAR2 protein expression in mouse tissues.

**Conclusion:**

We presented that stimulation of FFAR2 and FFAR4 may inhibit CRC cell viability and migration and that the FFAR2 and FFAR4 expression decreased in CRC can be restored by treatment with respective agonists, indicating new promising pharmacological targets in CRC treatment.

**Supplementary Information:**

The online version contains supplementary material available at 10.1007/s43440-024-00667-5.

## Introduction

Colorectal cancer (CRC) has one of the highest incidence rates of malignancies worldwide and ranks only behind lung and breast cancers in women and prostate cancer in men. It is the second leading cause of cancer-related mortality globally [1–3]. Approximately 70% of CRCs are sporadic, 3–5% are inherited, and in about 20–25% of patients, there is no identifiable inherited mutation, but there is a strong family history of CRC [4].

As the colonic epithelium progresses from normal to dysplastic, genetic changes accumulate over time, subsequently resulting in colon carcinoma [5]. Aberrant expression of many genes, such as *APC*, *SMAD4*, *TP53*,* PTEN*, and *KRAS* is involved in the pathophysiology of CRC [6, 7]. Recent data suggest that altered expression of free fatty acid receptors (FFARs) may also occur [8]. The FFAR family consists of four subtypes (FFAR1-4) which belong to the G protein-coupled receptor (GPCRs) superfamily. Categorically, FFAR1 (also termed GPR40) and FFAR4 (GPR120) are activated by long-chain fatty acids (LCFAs), whereas FFAR2 (GRP43) and FFAR3 (GPR41) - by short-chain fatty acids (SCFAs) [9, 10]. It is worth emphasizing that FFARs are engaged in multiple biological processes, such as insulin secretion [11], adipose tissue biology [12, 13], inflammation [14] as well as carcinogenesis [15]. FFARs have been implicated in diabetes, obesity, IBD, and various cancers, including breast and prostate cancer [16–22]. Recent evidence suggests that altered FFAR2 and FFAR4 expression may play a role in CRC pathogenesis [23]. Consequently, it has been proven that *FFAR2* gene expression is down-regulated in CRC patients [24]. Interestingly, reporting on *FFAR4* expression has changed over the years; earlier, data suggested an up-regulation of *FFAR4* in human tissue and CRC cell lines [25], while currently a down-regulation in *FFAR4* expression is seen in CRC patients’ tissue and animal models of CRC [26]. Independently, several groups of researchers have underlined the role of FFARs activation in CRC treatment [26–29].

The aim of this study was to evaluate the effect of FFAR2 and FFAR4 stimulation by selective synthetic agonists in in vitro and in vivo models of CRC and investigate their influence on FFAR expression.

## Materials & methods

### Cell lines culture

Human epithelial colon cell line, CCD 841 CoN (CRL-1790) and human colorectal adenocarcinoma cell line, SW-480 (CCL-228), purchased from American Type Culture Collection (ATCC), were cultured in the EMEM and RPMI media (Gibco^®^, Grand Island, NY, USA), respectively supplemented with 10% fetal bovine serum (FBS), 2 mM L-glutamine, 50 U/mL penicillin, and 50 µg/mL streptomycin. The cells were cultured in a humidified atmosphere of 5% CO_2_ at 37^o^C. All experiments were carried out between passages 5 to 15. Seeding quantities were such that the confluence of the control wells did not exceed 80% at the end of the experiment.

### Chemicals

All reagents, unless otherwise stated, were purchased from Merck (Poznan, Poland). Allosteric agonist 2-(4-chlorophenyl)-3-methyl-N-(thiazol-2-yl)butanamide (4-CMTB), activating FFAR2 [30] and 4-methoxy-N-(2,4,6-trimethylphenyl)benzenesulfonamide (GSK137647), a potent and selective FFAR4 agonist [31] were acquired from Tocris Bioscience (Bristol, UK, Cat. No. 4642 and 5257, respectively). For all in vivo tests, compounds were dissolved in 1% dimethyl sulfoxide.

### Cytotoxicity Assessment

MTT assay was used to measure the cytotoxicity in CCD 841 CoN and SW-480 cell lines [32]. Shortly, cells were seeded on a 96-well plate at the density of 6,000 cells/well and incubated for 24 h. Then, cells were incubated with 4-CMTB or GSK137647 (concentrations ranging from 10 to 200 µM) for 48 h. Next, 20 µL of MTT (5 mg/mL) was added to each well, and cells were incubated for an additional 2 h. Then, the medium was removed, 100 µL of DMSO was added to each well, and the plate was shaken for 10 min. Microplate reader (iMARK Microplate Reader, Biorad, Hertfordshire, UK) was used to measure optical density at 595 nm. A percentage of cells without treatment was used to express data.

### Invasion Assay

Matrigel BM matrix assay was used for measuring the capacity of cancer cells to motility, as previously described by Lewandowska et al. [33]. The cancer cell invasion was investigated using SW-480 cells. The test was performed in BioCoat Matrigel invasion chambers (24-well cell culture inserts containing an 8.0-µm PET membrane with a uniform layer of Matrigel; Becton Dickinson, Bedford, MA, USA, Cat. No. 354480), and the lower chamber contained cell culture medium with 10% FBS as a chemoattractant. The SW-480 cells were resuspended in an FBS-free medium with or without 4-CMTB (25 µM) or GSK137647 (50 µM) and loaded into the insert according to the manufacturer’s recommendations. The chambers were incubated for 48 h at 37 °C in a 5% CO_2_ atmosphere. The non-migrated cells in the upper chamber were then gently scraped away and adherent cells present on the PET membrane were stained with crystal violet. The five images of representative fields were taken with the EVOS FLoid Cell Imaging Station (Thermo Fisher Scientific, Bothell, WA, USA). The migrated cells were counted using NIH ImageJ analysis software.

### Migration Assay

Migration studies were completed as described in paragraph *Invasion assay*, using matrigel-free inserts: Falcon^®^ Permeable Support for 24-well Plate with 8.0 μm Transparent PET Membrane; Corning, Corning, NY, USA, Cat. No. 353097).

### Quantitative real-time polymerase chain reaction (qPCR)

Total RNA was extracted using TRIzol^®^ reagent (Invitrogen™, Carlsbad, CA, USA) and its concentration and purity were evaluated using a Calibri Microvolume Spectrophotometer (Biocompare, San Francisco, CA, USA). Total RNA (1 µg) was transcribed to cDNA with a high-capacity Reverse Transcriptase Kit (Life Technologies, Carlsbad, CA, USA). Quantitative assay of the gene expression was performed using labeled probes (Life Technologies, Carlsbad, CA, USA): *FFAR2* (Hs00271142_s1), *FFAR4* (Hs00699184_m1), and *HPRT1* (Hs1003267_m1) as endogenous control on the LightCycler 96 Instrument (Roche, Basel, Switzerland) with TaqMan Gene Expression Master Mix (Life Technologies, Carlsbad, CA, USA) according to the manufacturer’s protocol. All experiments were conducted in triplicate. The threshold cycle (Ct) values for studied genes were normalized to Ct values received for housekeeping *HPRT1* gene, and the relative expression of each gene was calculated using the 2^− ΔΔCt^ method.

### Protein isolation and western blot

Cell culture samples and tissue samples were incubated with 300 µL of the mammalian cell lysis buffer (50 mM Tris-HCl, pH 7.5; 1 mM EDTA, 150 mM NaCl; 0.1% SDS; 0.5% deoxycholic acid; and 1% Igepal CA-630) with protease-inhibitor cocktail. Subsequently, samples were homogenized using Precellys Evolution Homogenizer (Bertin Instruments, Paris, France) and centrifuged at 15,000 rpm for 15 min at 4^o^C. Then, supernatants containing proteins were transferred to new tubes and protein concentration was measured using the Pierce 660 nm protein assay (Thermo Scientific, Rockford, IL, USA) or Bicinchoninic Acid Protein Assay Kit (BCA; Sigma-Aldrich, Poznan, Poland). Electrophoresis was performed using a pre-made 4–20% SDS-PAGE gel (Bio-Rad, Warsaw, Poland) in a buffer containing 0.1% SDS, 192 mM glycine, 25 mM Tris, pH 8.3. Separated proteins were transferred onto PVDF membranes (pore size: 0.45 μm; Life Technologies, Carlsbad, CA, USA) using a semi-dry system with a transfer buffer containing 20% (v/v) methanol, 192 mM glycine, and 25 mM Tris, pH 8.3. Then, the PVDF membranes were blocked at RT for 1 h in 5% non-fat dry milk in phosphate-buffered saline (PBS) with Tween 20 (PBST; PBS, 0.1% Tween 20). The membranes were then incubated overnight at 4^o^C with specific primary antibodies diluted in 1% non-fat dry milk in PBST for immunodetection of proteins of interest. The primary rabbit FFAR2 polyclonal antibody (FFAR2-201AP, dil. 1:500; FabGennix, Thermo Fisher, USA), FFAR4 polyclonal antibody (E-AB-92023; dil. 1:1,000; Elabscience, Wuhan, China), transforming growth factor-β (TGF-β) polyclonal antibody (#3711, dil. 1:1,000; Cell Signaling Technology, Danvers, Massachusetts, USA), epithelial growth factor receptor (EGFR) polyclonal antibody (#2232, dil. 1:1,000; Cell Signaling Technology, Danvers, Massachusetts, USA), phosphorylated EGFR (pEGFR) polyclonal antibody (Tyr1068) (#2234, dil. 1:1,000; Cell Signaling Technology, Danvers, Massachusetts, USA), and HRP anti-beta Actin antibody (#ab49900, dil. 1:6,000, Abcam, Cambridge, England) were used. After the washing with PBST (5 times, 3 min), the membranes were incubated with appropriate secondary antibodies (anti-rabbit antibody (#A9169, dil: 1:6,000 Sigma-Aldrich, Poznan, Poland) and anti-mouse antibody (#AP200P, dil. 1:6,000, Merck, Poznan, Poland) for 1 h at RT. The bands were visualized using Super Signal West Pico western blotting substrate (Thermo Scientific, Rockford, IL, USA) as a substrate for the localization of HRP activity. Qualitative and quantitative analysis was performed by measuring integrated optical density using ImageLab v5.2.1 for the Windows™ program (Bio-Rad SA, Warsaw, Poland).

### Animals

Male Balb/C mice (provided by the Animal House of the University of Lodz, Łódź, Poland), weighing 22–26 g, were used in this study. The animals were kept in a room at constant temperature (22 ± 1 °C), humidity (70 ± 5%), and light/dark cycles conditions (12/12 h), in sawdust-coated transparent cages and had free access to chow and autoclaved water. An experimental group of mice was randomly assigned after one week of acclimatization. The experiments were conducted in accordance with the recommendations of the institution. Every effort was made to minimize the number of used animals in the experiments (according to the 3R role). All procedures were approved by the Local Ethical Committee for Animal Research in Lodz with the following number: 32/ŁB144/2019.

### Azoxymethane/Dextran sulfate Sodium-Induced Colitis-Associated Colorectal Cancer Model

Colitis-associated colorectal cancer (CACRC) was induced by a single intraperitoneal (*ip*) injection of azoxymethane (AOM, Sigma-Aldrich, Poznan, Poland; Cat. No. A5486) at the dose of 10 mg/kg of body weight (b.w.). One week later drinking water was substituted by 1.5% (w/v) dextran sodium sulfate (DSS, PanReac AppliChem, Darmstadt, Germany; Cat. No. A3261) solution. After one week, DSS was replaced by water for the next 2 weeks. This cycle was then repeated two more times. Body weight, health, and general behavior of each mouse were monitored at least twice per week. Animals that lost weight (> 20%), showed changes in their external appearance (hunched posture, dehydration signs), and showed changes in their behavior (labored breathing) were euthanized. At the end of the third week, 4-CMTB or GSK137647 (dissolved in 0.5% DMSO in 0.9% NaCl) was injected *ip* at the dose of 10 and 1 mg/kg, respectively, in the final volume 100 µL, every 3 days, until the end of 14 week. Animals without treatment received vehicle alone (0.5% DMSO in 0.9% NaCl). Untreated control mice received vehicle (0.9% NaCl) injection instead of AOM and were given water during the entire experimental period. The following experimental groups were distinguished: control (*n* = 7), AOM/DSS (*n* = 10), AOM/DSS + 4-CMTB (*n* = 10), and AOM/DSS + GSK137647 (*n* = 10). Overall number of animals used in the study was 37. The mortality rate was around 30–40% depending on the group. Therefore, in molecular studies, the following numbers of animals were used: control (*n* = 7), AOM/DSS (*n* = 7), AOM/DSS + 4-CMTB (*n* = 6), and AOM/DSS + GSK137647 (*n* = 7).

After 14 weeks, mice were euthanized and the macroscopic damage scoring was performed: spleen, liver, and colon were isolated, washed, and weighted. A longitudinal cut and opening of the colon were performed from the ileocecal junction to the anus. Under a dissecting microscope, the colon was examined macroscopically for tumor presence, subsequently, the colon was divided into two sections– first one for histological analysis and the second one for further molecular investigation.

### Histology

The colon tissue was rolled into Swiss rolls and fixed in 10% formalin. Five µm sections were cut and stained with hematoxylin and eosin. The sections were then examined under a microscope (Axio Imager A2 microscope, Carl Zeiss, Berlin, Germany). Five images from different parts of the intestine per magnification per animal in each experimental group were taken using ZEN (Blue 2.5 edition) imaging software. The microscopic score in AOM/DSS-induced CACRC included the following parameters: an inflammation (0–3, 0 = no inflammation, 1 = mild inflammation, 2 = inflammation, 3 = advanced inflammation), the morphology score (0–3, 0 = no changes in the intestinal wall architecture, 1 = elongated crypts, goblet cell present, small, few polyps, 2 = elongated crypts and/or absence of crypts, a partial loss of goblet cells, immune cell infiltration, numerous polyps, 3 = a loss of crypt and goblet cells, high infiltration with immune cells, adenoma), the ulceration score (0–1, 0 = absence, 1 = presence), and the lymph follicles score (0–1, 0 = absence, 1 = presence). Three representative images per experimental group are provided in the Supplementary section.

### Statistical analyses

The statistical analyses were performed with PRISM 8.0 (GraphPad Software Inc., La Jolla, CA, USA). Data is presented as mean ± SEM, or median ± ranges, as indicated in the figure legends. The number of independent experiments is given in the figure legends. The data were tested for normality using the Shapiro-Wilk test. Then, the statistical significance of differences between means was determined by parametric tests: one-way or two-way ANOVA followed by a post hoc multiple comparison Dunnett’s or Sidak’s test and a nonparametric test: Kruskal-Wallis test, followed by a post hoc multiple comparison Dunn’s. P values of < 0.05 were considered to be statistically significant.

## Results

### Stimulation of FFAR2 and FFAR4 by Synthetic agonists reduced cell growth

**Fig. 1:**
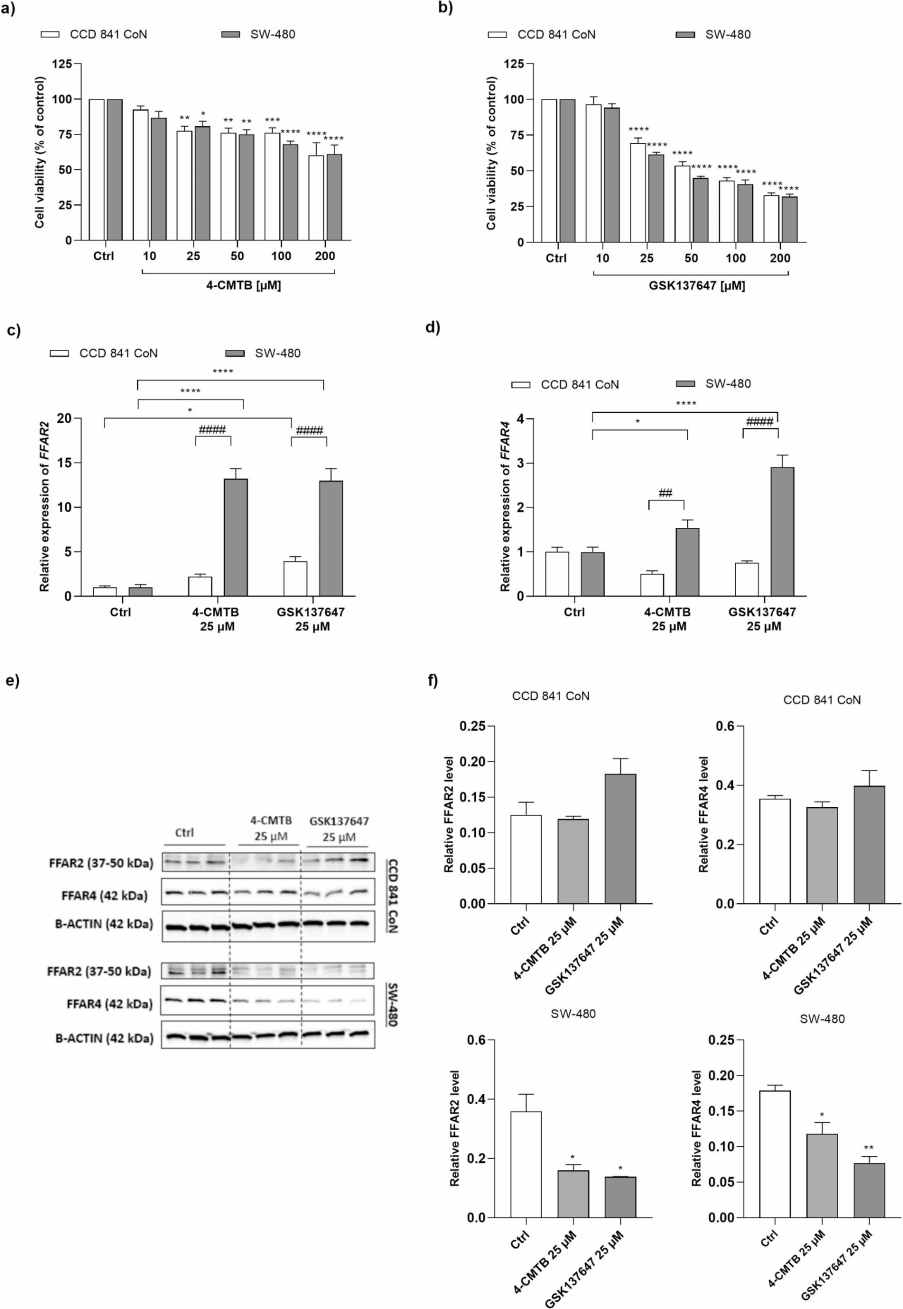
The influence of synthetic FFAR agonists on human intestinal epithelial CCD 841 CoN cells and human cancer intestinal epithelial SW-480 cell in vitro. Cell viability measured by MTT assay after 48 h treatment with various concentrations of the FFAR2 agonist, 4-CMTB **(a)** and the FFAR4 agonist, GSK137647 **(b)**. Expression of *FFAR2***(c)** and *FFAR4***(d)** genes in CCD 841 CoN cells and SW-480 cells determined using a real-time qPCR. Representative western blot images demonstrating the expression of the analyzed proteins in the whole cell lysates and changes in the FFAR protein concentration after 48 h exposure to FFARs agonists **(e).** Quantification of protein level by densitometry calculated by Image Lab **(f)**. Each value represents mean ± SEM, *n* = 3 independent experiments (each experiment was carried out in six repetitions) for MTT assay and *n* = 3 samples per group for real-time qPCR and western blot. Data were analyzed with two-way ANOVA (a, b, c, d) and one-way ANOVA (e), followed by Dunnett’s or Sidak’s post hoc.*p < 0.05, **p < 0.01, ***p < 0.001, ****p < 0.0001 as compared to control group; and ##*p* < 0.01, ####*p* < 0.0001, SW-480 vs. CCD 841 CoN cells treated the same way. Abbreviations: Ctrl: control; FFAR: free fatty acid receptor; FFAR2: free fatty acid receptor type 2; FFAR2: free fatty acid receptor type 4

FFAR2 agonist, 4-CMTB, and FFAR4 agonist, GSK137647 significantly decreased both non-cancer (CCD 841 CoN) and cancer (SW-480) cell growth at the concentrations 10–200 µM as compared to untreated cells (Fig. 1a-b, S1a-b). Two-way ANOVA revealed following results: for 4-CMTB (effect of factor 1: F_5,30_=20.38, *p* = 0.0001; effect of factor 2: F_1,30_=0.5166, *p* = 0.4778; interaction: F_5,30_=0.5505, *p* = 0.7367) and for GSK137647 (effect of factor 1: F_5,30_=207.5, *p* = 0.0001; effect of factor 2: F_1,30_=5.596, *p* = 0.0247; interaction: F_5,30_=0.8621, *p* = 0.5178). To note, the cytotoxicity of GSK137647 on both cell lines was higher than the cytotoxicity of 4-CMTB.

### *FFAR2* and *FFAR4* gene expressions were increased after treatment with 4-CMTB and GSK137647 in SW-480 cells vs. CCD 841 CoN

The lowest concentration which significantly reduced CRC cells viability, i.e. 25 µM for both synthetic compounds (as shown in Fig. 1a-b, S1) was chosen to investigate their effect on *FFAR* expression. Two-way ANOVA revealed that both 4-CMTB and GSK1376547 significantly increased *FFAR2* gene expression (effect of factor 1: F_2, 12_=55.31, *p* = 0.0001; effect of factor 2: F_1, 12_=109.3 *p* = 0.0001; interaction: F _2,12_=28.08, *p* = 0.0001). Both tested compounds significantly higher *FFAR2* gene expression in SW-480 cells compared to untreated cells (in both treatments, *p* = 0.0001). Interestingly, only the FFAR4 agonist, GSK137647 but not the FFAR2 agonist, 4-CMTB significantly elevated *FFAR2* gene expression in CCD 841 CoN cells (*p* = 0.0369). To note, when comparing changes in FFAR2 gene expression between CRC cells and non-CRC, an 11-fold and 9-fold increase after 4-CMTB and GSK137647 treatment, respectively, was observed in SW-480 vs. CCD 841 CoN cells (*p* = 0.0001) (Fig. 1c, S1c).

Analysis by two-way ANOVA exposed statistically significant differences between *FFAR4* gene expression and treatment groups (effect of factor 1: F_2, 12_=19.76, *p* = 0.0002; (effect of factor 2: F_1, 12_=74.39, *p* = 0.0001, interaction: F _2,12_=25.65, *p* = 0.0001). 4-CTMB and GSK137647 significantly increased *FFAR4* gene expression in SW-480 cells compared to untreated cells (*p* = 0.0462 and *p* = 0.0001, respectively). In addition, *FFAR4* gene expression was 3-fold and 4-fold higher after 4-CMTB and GSK137647 treatment, respectively, when SW-480 were compared vs. CCD 841 CoN cells (*p* = 0.0012 and *p* = 0.0001, respectively)(Fig. 1d, S1d).

### 4-CMTB and GSK137647 decreased FFAR2 and FFAR4 protein levels in CRC cells

Western blot analysis, followed by one-way ANOVA showed that there was no difference in FFAR2 and FFAR4 protein levels between CCD 841 CoN cells treated with 4-CMTB or GSK137647 and untreated controls (F_2,6_=4.649, *p* = 0.0603 and F_2,6_=1.261, *p* = 0.3490). Surprisingly, one-way ANOVA revealed that FFAR2 and FFAR4 protein levels were decreased in 4-CMTB or GSK137647-treated SW-480 cells as compared to untreated controls (F_2,5_=9.136, *p* = 0.0214 and F_2,6_=19.61, *p* = 0.0023). 4-CMTB or GSK137647 significantly lower FFAR2 protein level (*p* = 0.0259 and *p* = 00269, respectively), and FFAR4 protein level (*p* = 0.0176 and *p* = 0.0014, respectively) in CRC cells (Fig. 1e-f, S2).

### FFAR2 and FFAR4 agonists reduced the metastatic potential of CRC cells

**Fig. 2:**
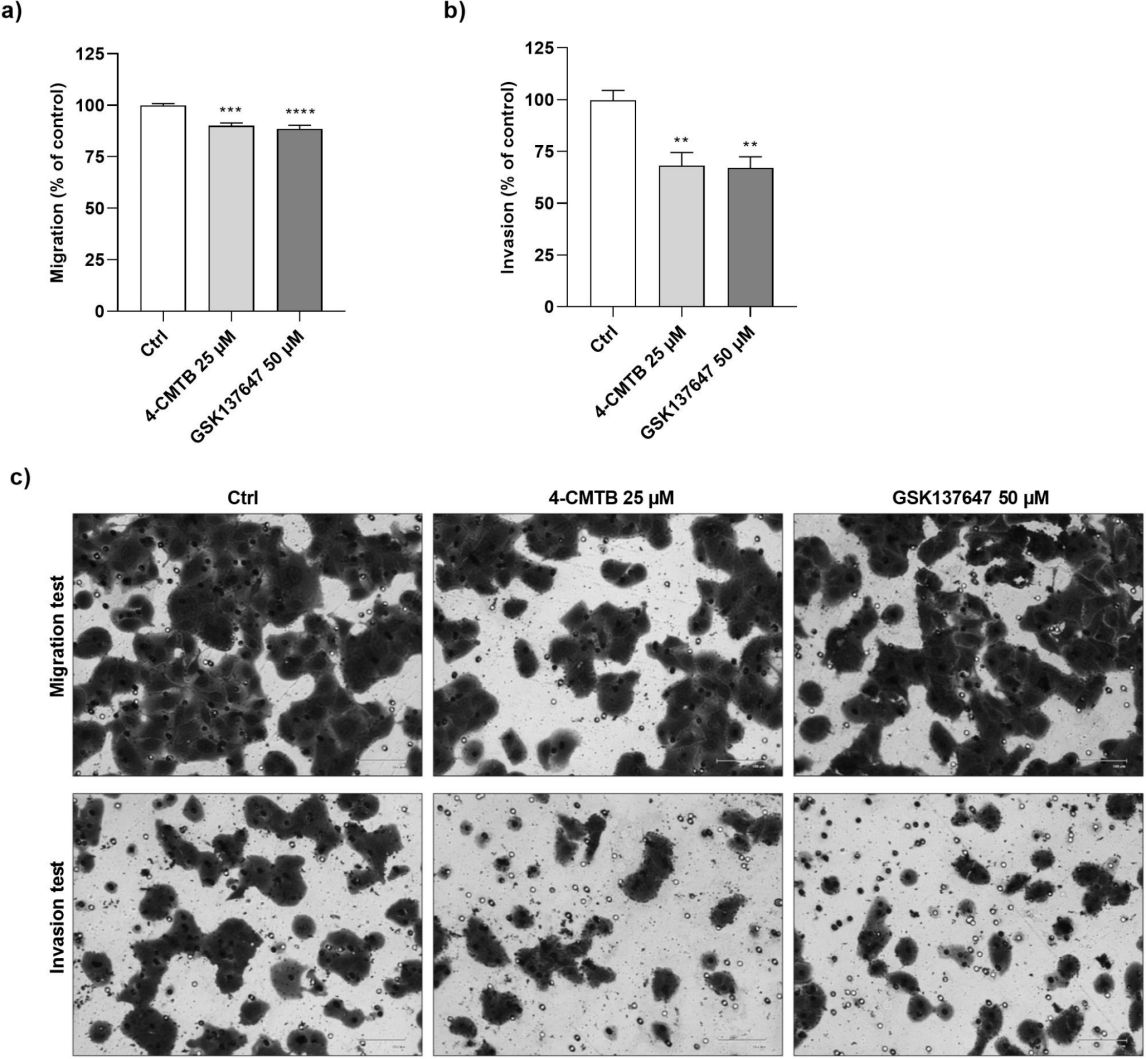
Effects of 4-CMTB and GSK137647 on the SW-480 cell migration and invasion. SW-480 cell migration **(a)** and invasion **(b)** were determined after 48 h incubation of cells on inserts with indicated concentrations of compounds. Representative images **(c)** of cells after 48 h incubation. Each value represents mean ± SEM, *n* = 3 independent experiments (each experiment was carried out in duplicate). Data were analyzed with one-way ANOVA test, followed by Dunnett’s post hoc. Significance of differences between means: **p < 0.01, ***p < 0.001, ****p < 0.0001 versus control (untreated cells). Abbreviations: Ctrl: control

Concentrations 25 µM and 50 µM for 4-CMTB and GSK137647, respectively, were selected in preliminary experiments with migration tests (data not shown). The wound healing assay, followed by one-way ANOVA showed statistically significant differences between migration and invasion and treatment groups (F_2,15_=20.89, *p* = 0.0001 and F_2,15_=11.06, *p* = 0.0011, respectively). 4-CMTB and GSK137647 significantly decreased (by approx. 20%) the migration of SW-480 cells as compared to untreated cells (*p* = 0.0002 and *p* = 0.0001, respectively)(Fig. 2a). Furthermore, FFAR2 and FFAR4 agonists significantly reduced SW-480 cell invasion by around 30% measured in matrigel bio-coated chambers (*p* = 0.0022 and *p* = 0.0016, respectively)(Fig. 2b). Migration and invasion of CRC cells were decreased after FFAR2 and FFAR4 agonist treatments (Fig. 2c, S3a-b) suggesting that FFARs stimulation inhibits metastatic properties of CRC cells.

### Stimulation of FFAR2 and FFAR4 by Synthetic agonists did not affect AOM-DSS-Induced CACRC in mice

**Fig. 3:**
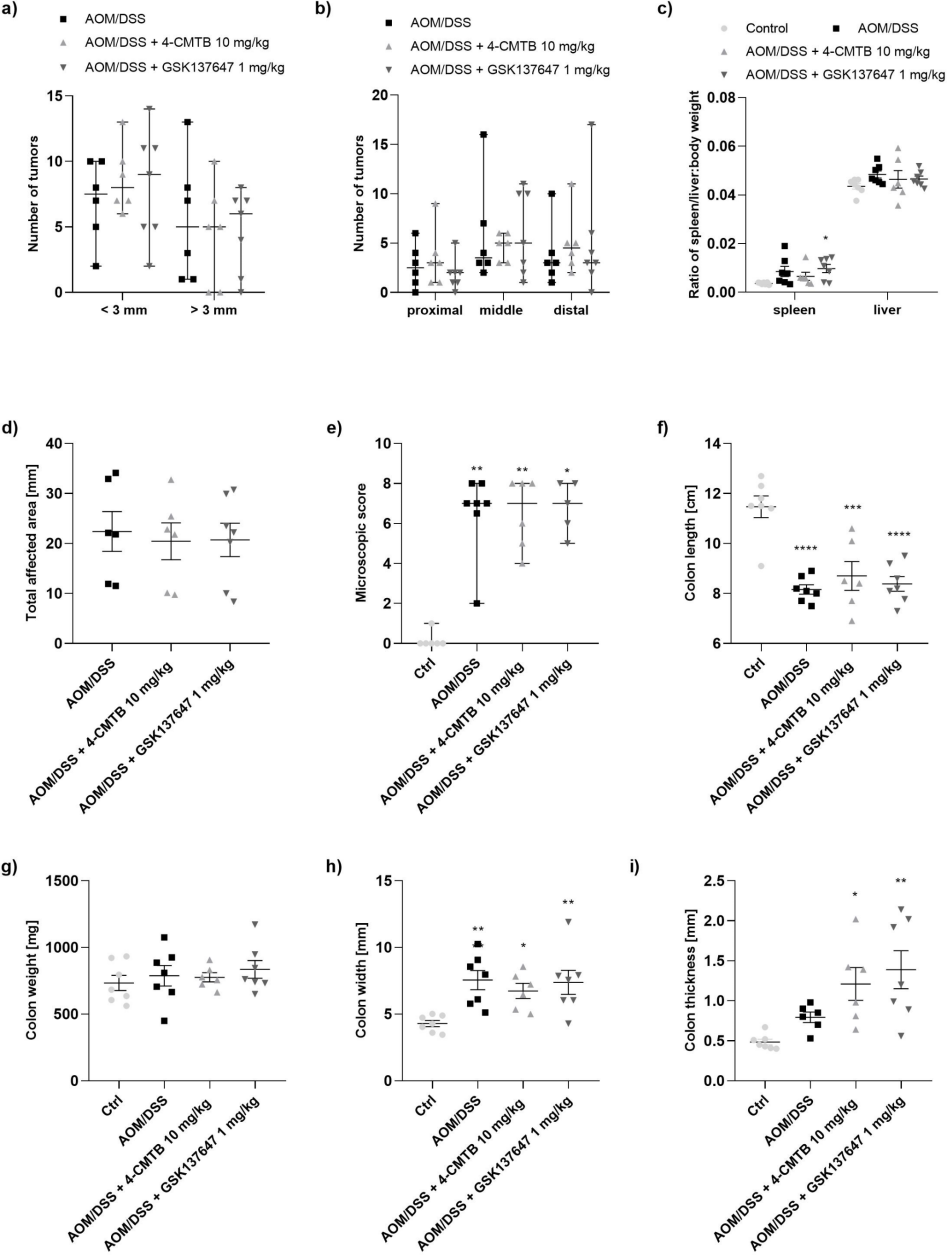
The effect of 4-CMTB and GSK137647 in the mouse model of AOM/DSS-induced colitis associated colorectal cancer indicated by the following parameters: **(a**,** b)** tumor number, **(c)** spleen and liver/body weight ratio, **(d)** total affected area, **(e)** microscopic score, **(f)** colon length, **(g)** colon weight, **(h)** colon width and **(i)** colon thickness. Data were analyzed with Kruskal-Wallis test, followed by Dunn’s post hoc (a, b, e) and one-way or two-way ANOVA test (c, d, f, g, h, i), followed by Dunnett’s post hoc. Data represent median ± ranges (a, b, e) or mean ± SEM (c, d, f, g, h, i) for *n* = 6–7 mice per group. *p < 0.05, **p < 0.01, ***p < 0.001, ****p < 0.0001 as compared to control. Abbreviations: AOM: azoxymethane; Ctrl: control; DSS: dextran sulfate sodium

To investigate the anti-cancer effect of FFAR2 and FFAR4 stimulation in the mouse model mimicking CACRC, an AOM/DSS-induced CACRC model was used. Based on the available literature, 4-CMTB at the dose of 10 mg/kg b.w. and GSK137647 at the dose of 1 mg/kg b.w. were injected three times/week. Administration of AOM and DSS developed CACRC seen in the number of tumors (H = 6.433, *p* = 0.0752)(Fig. 3a-b) assessed by Kruskal-Wallis test, the ratio of spleen/liver: b.w. (effect of factor 1: F_1,46_=960.3, *p* = 0.0001; effect of factor 2: F_3,46_=3.283, *p* = 0.029; interaction: F_3,46_=0.3808, *p* = 0.7673)(Fig. 3c, S4c) by two-way ANOVA, the total affected area (F_2,16_=0.08079, *p* = 0.9228)(Fig. 3d, S4d) by one-way ANOVA, microscopic score (H = 13.48, *p* = 0.0037)(Fig. 3e, S4e, S5) by Kruskal-Wallis, colon length (F_3,23_=16.63, *p* = 0.0001), weight (F_3,23_=0.4672, *p* = 0.7080), width (F _3,23_=5.366, *p* = 0.006), and thickness (F_3,23_=6.536, *p* = 0.0025)(Fig. 3f-i, S4f-i) by one-way ANOVA. Unfortunately, injection of FFAR2 or FFAR4 agonist did not cause any improvement in the disease score.

### 4-CMTB increased Ffar2 protein level, whereas GSK1375647 affected Tgfβ level in the CACRC mouse Colon

**Fig. 4:**
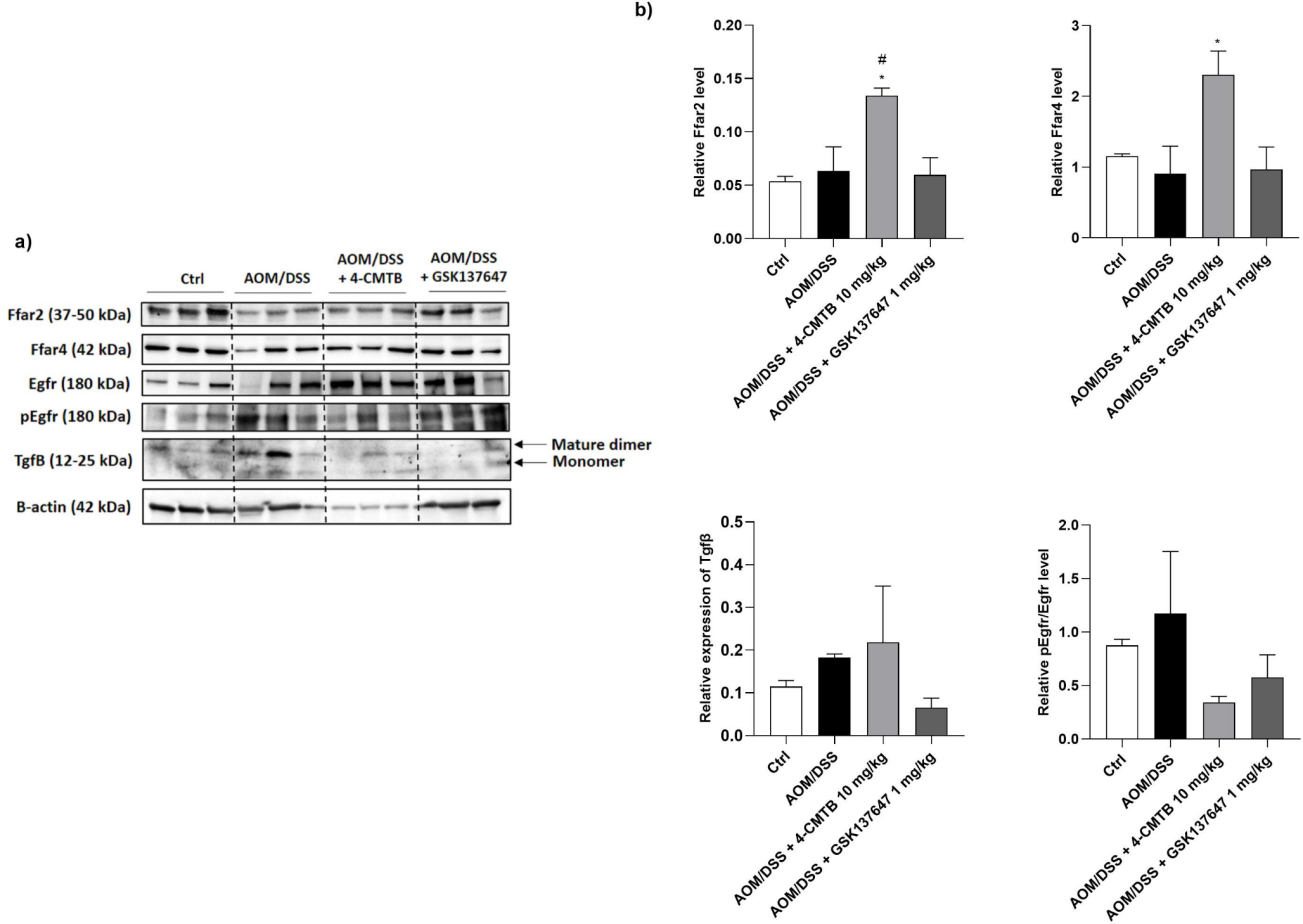
Representative blots of Ffar2, Ffar4, Egfr, pEgfr, Tgfβ and β-actin for control, AOM/DSS and AOM/DSS + 4-CMTB and GSK137647-treated group **(a)** and densitometry of indicated proteins **(b)** in the mouse colon. Data were analyzed with one-way ANOVA test, followed by Dunnett’s post hoc. Data represent mean ± SEM for *n* = 6 samples per group. *p < 0.05 as compared to control, #*p* < 0.05, ##*p* < 0.01 as compared to AOM/DSS group. Abbreviations: AOM: azoxymethane; Ctrl: control; DSS: dextran sulfate sodium; FFAR2: free fatty acid receptor type 2; FFAR4: free fatty acid receptor type 4; Egfr: epithelial growth factor receptor; pEgfr: phosphorylated epithelial growth factor receptor; Tgfβ: transforming growth factor β

Analysis by one-way ANOVA exposed statistically significant differences between Ffar2 and Ffar4 protein levels and treatment groups (F_3,8_=6.747, *p* = 0.0139 and F_3,8_= 4.671, *p* = 0.0361). CACRC mice treated with 4-CMTB at the dose of 10 mg/kg b.w. had significantly elevated Ffar2 and Ffar4 protein levels compared to untreated mice with CACRC (*p* = 0.0222 and *p* = 0.0288, respectively). Moreover, a slightly lower level of pEgfr/Egfr was detected in the colon of 4-CMTB-treated CACRC mice than in CACRC mice without treatment (Fig. 4a-b, S6).

GSK137647 did not alter any FFAR protein level in the CACRC mouse colon; however, this compound faintly decreased transforming growth factor β (Tgfβ) expression in the colon of CACRC mice compared to CACRC mice without treatment (Fig. 4a-b, S5).

## Discussion

Treatment of GI disorders with complex pathophysiology, as in the case of CRC is still a huge challenge for clinicians, researchers, and, above all, for patients. Here, taking into consideration previous findings suggesting the involvement of FFARs in carcinogenesis, we investigated the effect of FFAR2 and FFAR4 stimulation by synthetic ligands in in vitro and in vivo models of CRC. The obtained results indicated that 4-CMTB (a FFAR2 agonist) and GSK137647 (a FFAR4 agonist) reduced the CRC cell growth and their capability to migration and invasion. Importantly, both synthetic compounds increased *FFAR2* and *FFAR4* gene expression in CRC cells. Although neither 4-CMTB nor GSK137647 influenced the course of AOM/DSS-induced CACRC in mice, the protein expression analysis revealed changes in FFAR2 level after treatment with 4-CMTB.

Firstly, we discovered that an allosteric FFAR2 agonist, 4-CMTB significantly decreased SW-480 growth as well as their migration and invasion potentials in connection with an increased *FFAR2* gene expression. Our preliminary data showed that SW-480 cells have significantly lower *FFAR2* gene expression compared to colon epithelium cells, CCD 481 CoN (*p* < 0.05) (data not shown). In line, Tang et al. [24] also demonstrated that FFAR2 expression is extremely low in many CRC cell lines, including SW-480, SW-620, Caco-2, HCT116, HCT8, and HT-29. Fascinatingly, we observed that 4-CMTB also elevated *FFAR4* gene expression in the CRC cells. To note, we did not observe these trends in protein levels. Obtained data suggest that 4-CMTB can modulate *FFAR2* and even *FFAR4* gene expression and in consequence influence CRC development and progression.

Secondly, we exposed that 4-CMTB significantly increased FFAR2 and slightly FFAR4 protein levels in colon tissue samples from AOM/DSS mice. Interestingly, our preliminary data presented that *FFAR2* gene expression is decreased in mouse tissue with AOM/DSS-induced CACRC vs. control mice tissue without CACRC (data not shown), however, no significant differences in FFAR2 protein levels between groups were observed. The literature claims that FFAR2 immunoreactivities are evident in nearly all normal colon tissue samples, and successively reduced, or even lost in colon cancer tissues in a grade-dependent manner [24]. Unfortunately, our FFAR2 agonist did not influence the course of AOM/DSS-induced CACRC in in vivo experiments. Untreated CACRC mice and mice treated with 4-CMTB had a similar number of tumors and other cancer parameters, like total affected area or colon length, weight, and thickness. Literature shows that knocking out (KO) of FFAR2 promoted colon adenoma development in the Apc^Min/+^/DSS mice and adenocarcinoma progression in the AOM/DSS mice [34, 35]. Lavoie et al. [27] discovered that FFAR2-deficient mice developed colon tumors by reducing gut barrier integrity, increasing tumor bacterial load, exhausting CD8 + T cells, and overactivating dendritic cells (DCs), frequently leading to their destruction. Then, they observed that an FFAR2 agonist reduced the number of colon tumors, and decreased the frequency of IL27 + DCs in tumors of ApcMin/+/DSS mice [27]. There may be several reasons for the failure to obtain the anticancer effect of the FFAR2 agonist in mice, i.e. too low a dose of the used compound and infrequent solution injection, as well as improper route of administration, mouse strain, and CRC model.

Thirdly, we observed that *FFAR4* gene expression is also reduced in SW-480 vs. CCD 841 CoN cells (data not shown), however, it can be restored by its synthetic, selective agonist. Consequently, in this manuscript, we discuss the anti-cancer properties of the FFAR4 agonist, GSK137647. In the beginning, we established that this compound significantly reduced SW-480 growth. Furthermore, migration and invasion of CRC cells were decreased by around 20 and 40%, respectively, after cell treatment with GSK137647. Finally, gene expression analysis revealed that GSK137647-treated cells had increased *FFAR2* and *FFAR4* expression. As shown in recently published studies, restoration of FFAR4 expression is crucial for anti-cancer effect because CRC cells with FFAR4 knockdown indicate an increase in proliferation [26]. Moreover, the loss of epithelial FFAR4 increased intestinal permeability, microbiota translocation, and dysbiosis in AOM/DSS mice [26]. Subsequently, FFAR4 expression is also decreased with increasing CRC grade [26]. Although in our experiment, the FFAR4 agonist did not affect the number of tumors and FFARs protein levels in AOM/DSS-induced CACRC in mice, the decrease in TGF-β level was seen after treatment with GSK137647. TGF-β plays a critical role in tumorigenesis and promotes CRC development by stimulating epithelial-to-mesenchymal transition [36], suggesting that GSK137647 may inhibit CRC progression. Consequently, our group discovered the anti-inflammatory properties of this FFAR4 agonist in colitis [37]. In vitro studies showed that GSK137647 reduced the level of nitric oxide (NO) secreted by macrophages, RAW264.7-treated with LPS as well as decreased IL-6 production in response to the mixture of cytokines and LPS in Caco-2 cells. Furthermore, the *ip* administration of GSK137647 at the dose of 1 mg/kg b.w. attenuated colonic injuries induced by DSS and TNBS in mouse models of inflammatory bowel diseases (IBD) [37]. Similar results were obtained by another research team from China [19]. FFAR4 agonist significantly lowered NO production in RAW264.7 cells treated with free fatty acids (FFAs) and mediated M2 macrophage polarization. Moreover, FFAR4 activation by GSK1376647 reduced the accumulation of lipids causing an amelioration of DSS-induced ulcerative colitis (UC) [19]. The data discussed above, together with the fact that colonic inflammation may underlie CRC development, strongly suggest that FFAR4 activation should be investigated further in the context of its anti-cancer effect in CRC.

Several factors need to be taken into consideration in terms of the study’s limitations. Among genetic, xenografts, and chemical models of CRC, we selected the AOM/DSS-induced CACRC, which is commonly used to study carcinogenesis associated with chronic inflammation of the large intestine. It is worth to emphasize that exposure and doses of AOM and DSS vary significantly across different studies, therefore the obtained results can be difficult to analyze and compare [38]. In addition, one needs to observe the method of stimulation of FFARs selected for this study. These receptors are activated by FFAs which occur in our body mostly from diet, however, the selectivity of these natural compounds is difficult to predict. Using synthetic and selective agonists with high purity was necessary to ensure that selected receptors are stimulated effectively. 4-CMTB and GSK137647 were nominated to this study; nevertheless, still, few other FFAR agonists are available, like TUG841, an FFAR4 agonist which is widely investigated in numerous conditions, including diabetes, atherosclerosis, and lung cancer [28, 39, 40]. It needs to be taken into consideration that the use of TUG841 or non-selective natural agonists of FFARs could produce different effects than the ones reported herein; we are in due course of these investigations.

To sum up, the failure of 4-CMTB and GSK137647 to impact the AOM/DSS model suggests that different dosing regimens or CRC models should be explored. Future studies may also consider combining FFAR agonists with existing CRC therapies to evaluate potential synergistic effects.

## Conclusion

In our study, for the first time, we demonstrated that 4-CMTB and GSK137647 may restore *FFAR2* and *FFAR4* expression and then positively influence in vitro and in vivo models of CRC. Moreover, we provided experimental evidence that FFAR2 and FFAR4 may be therapeutic targets for CRC and CACRC. While further investigation is needed, our study highlights the potential of FFAR2 and FFAR4 agonists as future pharmacological targets for CRC treatment.

## Electronic supplementary material

Below is the link to the electronic supplementary material.

Supplementary Material 1

## Data Availability

No datasets were generated or analysed during the current study.

## Abbreviations

AOM: Azoxymethane
CACRC: Colitis-associated colorectal cancer
CRC: Colorectal cancer
DMSO: Dimethyl sulfoxide
DSS: Dextran sodium sulfate
EGF: Epithelial growth factor
EGFR: Epithelial growth factor receptor
FBS: Fetal bovine serum
FFAs: Free fatty acids
FFAR2: Free fatty acid receptor type 2
FFAR4: Free fatty acid receptor type 4
HTAB: Hexadecyltrimethylammonium bromide
IBD: Inflammatory bowel disease
pEGFR: Phosphorylated epithelial growth factor receptor
TGF-β: Transforming growth factorβ
UC: Ulcerative colitis
