# Supplementary material for: Synthetic free fatty acid receptor (FFAR) 2 agonist 4-CMTB and FFAR4 agonist GSK13764 inhibit colon cancer cell growth and migration and regulate FFARs expression in in vitro and in vivo models of colorectal cancer
Source: Pharmacol Rep. 2024 Oct 21;76(6):1403–14. doi: 10.1007/s43440-024-00667-5 (PMC11582145; doi:10.1007/s43440-024-00667-5)
Supplement: Supplementary file 1 — Supplementary Material 1 [file 43440_2024_667_MOESM1_ESM.docx]

**Tab. S1** Exact statistical values for Fig. 1.

|  | **Significance** | **Exact P-value** |
| --- | --- | --- |
| **a) Two-way ANOVA**  **Dunnett's multiple comparisons test** |  |  |
| **F_5, 30_ = 20.38** |  |  |
| CCD-841 CoN cell line |  |  |
| Ctrl vs. 10 | ns | 0.4918 |
| Ctrl vs. 25 | ** | 0.0052 |
| Ctrl vs. 50 | ** | 0.0028 |
| Ctrl vs. 100 | *** | 0.0006 |
| Ctrl vs. 200 | **** | <0.0001 |
| SW-480 cell line |  |  |
| Ctrl vs. 10 | ns | 0.1586 |
| Ctrl vs. 25 | * | 0.0196 |
| Ctrl vs. 50 | ** | 0.0019 |
| Ctrl vs. 100 | **** | <0.0001 |
| Ctrl vs. 200 | **** | <0.0001 |
| **b) Two-way ANOVA**  **Dunnett's multiple comparisons test** |  |  |
| **F_5, 30_ = 207.5** |  |  |
| CCD-841 CoN cell line |  |  |
| Ctrl vs. 10 | ns | 0.8223 |
| Ctrl vs. 25 | **** | <0.0001 |
| Ctrl vs. 50 | **** | <0.0001 |
| Ctrl vs. 100 | **** | <0.0001 |
| Ctrl vs. 200 | **** | <0.0001 |
| SW-480 cell line |  |  |
| Ctrl vs. 10 | ns | 0.5231 |
| Ctrl vs. 25 | **** | <0.0001 |
| Ctrl vs. 50 | **** | <0.0001 |
| Ctrl vs. 100 | **** | <0.0001 |
| Ctrl vs. 200 | **** | <0.0001 |
| **c) Two-way ANOVA**  **Dunnett's multiple comparisons test** |  |  |
| **F _2, 12_ = 55.31** |  |  |
| CCD-841 CoN cell line |  |  |
| Ctrl vs. 4-CMTB | ns | 0.4595 |
| Ctrl vs. GSK137647 | * | 0.0369 |
| SW-480 cell line |  |  |
| Ctrl vs. 4-CMTB | **** | <0.0001 |
| Ctrl vs. GSK137647 | **** | <0.0001 |
| **Sidak's multiple comparisons test** |  |  |
| **F_1, 12_ = 109.3** |  |  |
| Ctrl | ns | >0.9999 |
| 4-CMTB | **** | <0.0001 |
| GSK137647 | **** | <0.0001 |
| **d) Two-way ANOVA**  **Dunnett's multiple comparisons test** |  |  |
| **F_2, 12_ = 19.76** |  |  |
| CCD 841 CoN cell line |  |  |
| Ctrl vs. 4-CMTB | ns | 0.07 |
| Ctrl vs. GSK137647 | ns | 0.4297 |
| SW-480 cell line |  |  |
| Ctrl vs. 4-CMTB | * | 0.0462 |
| Ctrl vs. GSK137647 | **** | <0.0001 |
| **Sidak's multiple comparisons test** |  |  |
| **F _1, 12_ = 74.39** |  |  |
| CCD 841 CoN vs. SW-480 |  |  |
| Ctrl | ns | >0.9999 |
| 4-CMTB | ** | 0.0012 |
| GSK137647 | **** | <0.0001 |

Abbreviations: Ctrl: control, ns: not significant.


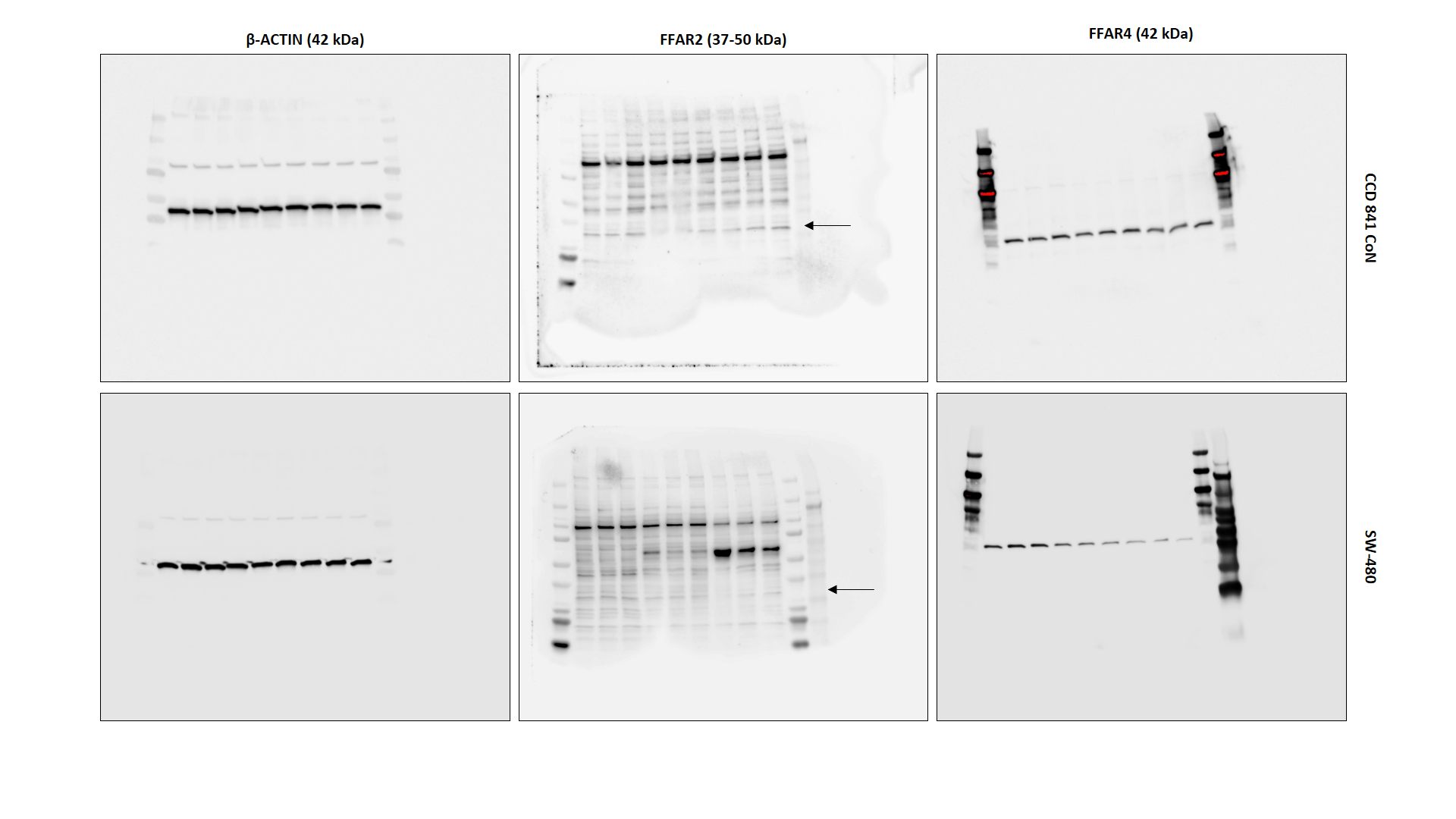


**Fig. S2** Unprocessed representative blots of β-actin, Ffar2, and Ffar4 from in vitro experiment.

Abbreviations: FFAR2: free fatty acid receptor type 2; FFAR4: free fatty acid receptor type 4.


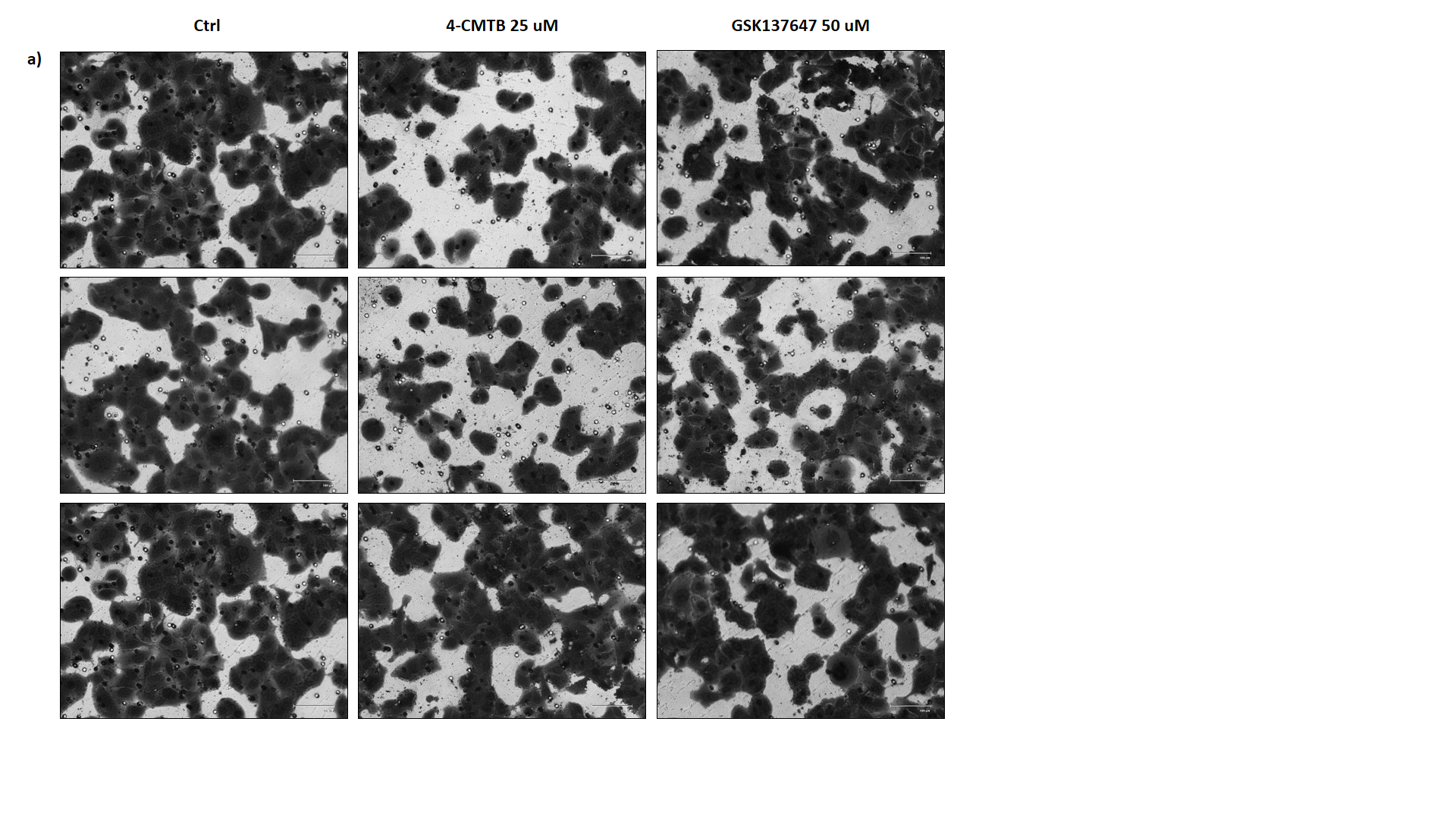


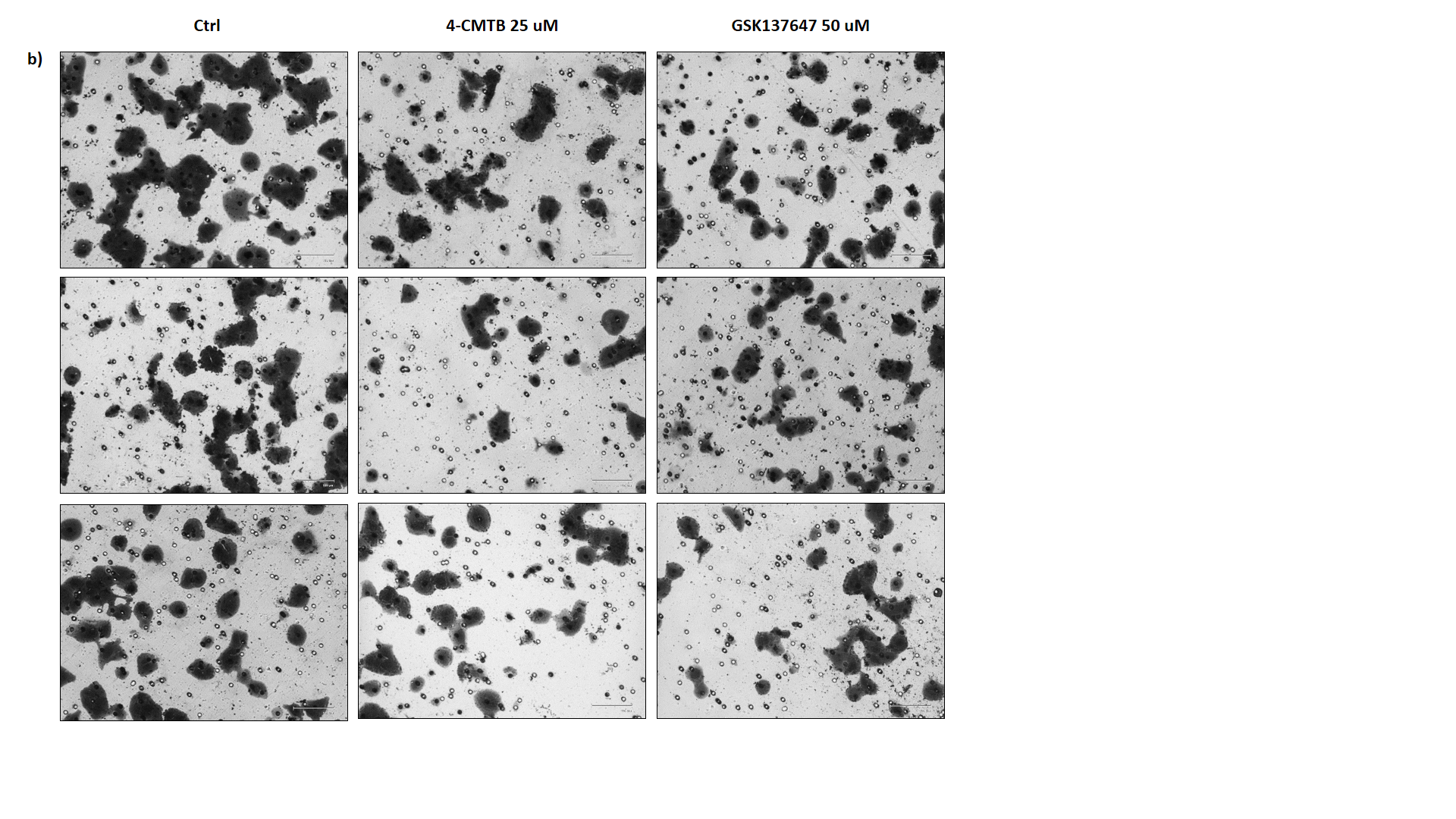


**Fig. S3** Representative images of migrated cells after 48h incubation in the migration (a) and invasion (b) test. Three photos per one experimental group.

Abbreviations: Ctrl: control.

**Tab. S4** Exact statistical values for Fig. 3.

|  | **Significance** | **Exact P-value** |
| --- | --- | --- |
| **c) Two-way ANOVA**  **Dunnett’s multiple comparison test** |  |  |
| Spleen |  |  |
| Ctrl vs. AOM/DSS | ns | 0.1300 |
| Ctrl vs. AOM/DSS + 4-CMTB 10 mg/kg | ns | 0.5373 |
| Ctrl vs. AOM/DSS + GSK137647 1 mg/kg | * | 0.0448 |
| liver |  |  |
| Ctrl vs. AOM/DSS | ns | 0.1361 |
| Ctrl vs. AOM/DSS + 4-CMTB 10 mg/kg | ns | 0.5586 |
| Ctrl vs. AOM/DSS + GSK137647 1 mg/kg | ns | 0.4832 |
| **d) One-way ANOVA**  **Dunnett’s multiple comparisons test** |  |  |
| AOM/DSS vs. AOM/DSS + 4-CMTB 10 mg/kg | ns | 0.9052 |
| AOM/DSS vs. AOM/DSS + GSK137647 1 mg/kg | ns | 0.9242 |
| **e) Kruskal-Wallis test**  **Dunn’s multiple comparisons test** |  |  |
| Ctrl vs. AOM/DSS | ** | 0.0056 |
| Ctrl vs. AOM/DSS + 4-CMTB 10 mg/kg | ** | 0.0094 |
| Ctrl vs. AOM/DSS + GSK137647 1 mg/kg | * | 0.0110 |
| **f) One-way ANOVA**  **Dunnett’s multiple comparisons test** |  |  |
| Ctrl vs. AOM/DSS | **** | 0.0001 |
| Ctrl vs. AOM/DSS + 4-CMTB 10 mg/kg | *** | 0.0001 |
| Ctrl vs. AOM/DSS + GSK137647 1 mg/kg | **** | 0.0001 |
| **g) One-way ANOVA**  **Dunnett’s multiple comparisons test** |  |  |
| Ctrl vs. AOM/DSS | ns | 0.8683 |
| Ctrl vs. AOM/DSS + 4-CMTB 10 mg/kg | ns | 0.9367 |
| Ctrl vs. AOM/DSS + GSK137647 1 mg/kg | ns | 0.5176 |
| **h) One-way ANOVA**  **Dunnett’s multiple comparisons test** |  |  |
| Ctrl vs. AOM/DSS | ** | 0.0048 |
| Ctrl vs. AOM/DSS + 4-CMTB 10 mg/kg | * | 0.0461 |
| Ctrl vs. AOM/DSS + GSK137647 1 mg/kg | ** | 0.0075 |
| **i) One-way ANOVA**  **Dunnett’s multiple comparisons test** |  |  |
| Ctrl vs. AOM/DSS | ns | 0.4245 |
| Ctrl vs. AOM/DSS + 4-CMTB 10 mg/kg | * | 0.0133 |
| Ctrl vs. AOM/DSS + GSK137647 1 mg/kg | ** | 0.0015 |

Abbreviations: Ctrl: control, ns: not significant.


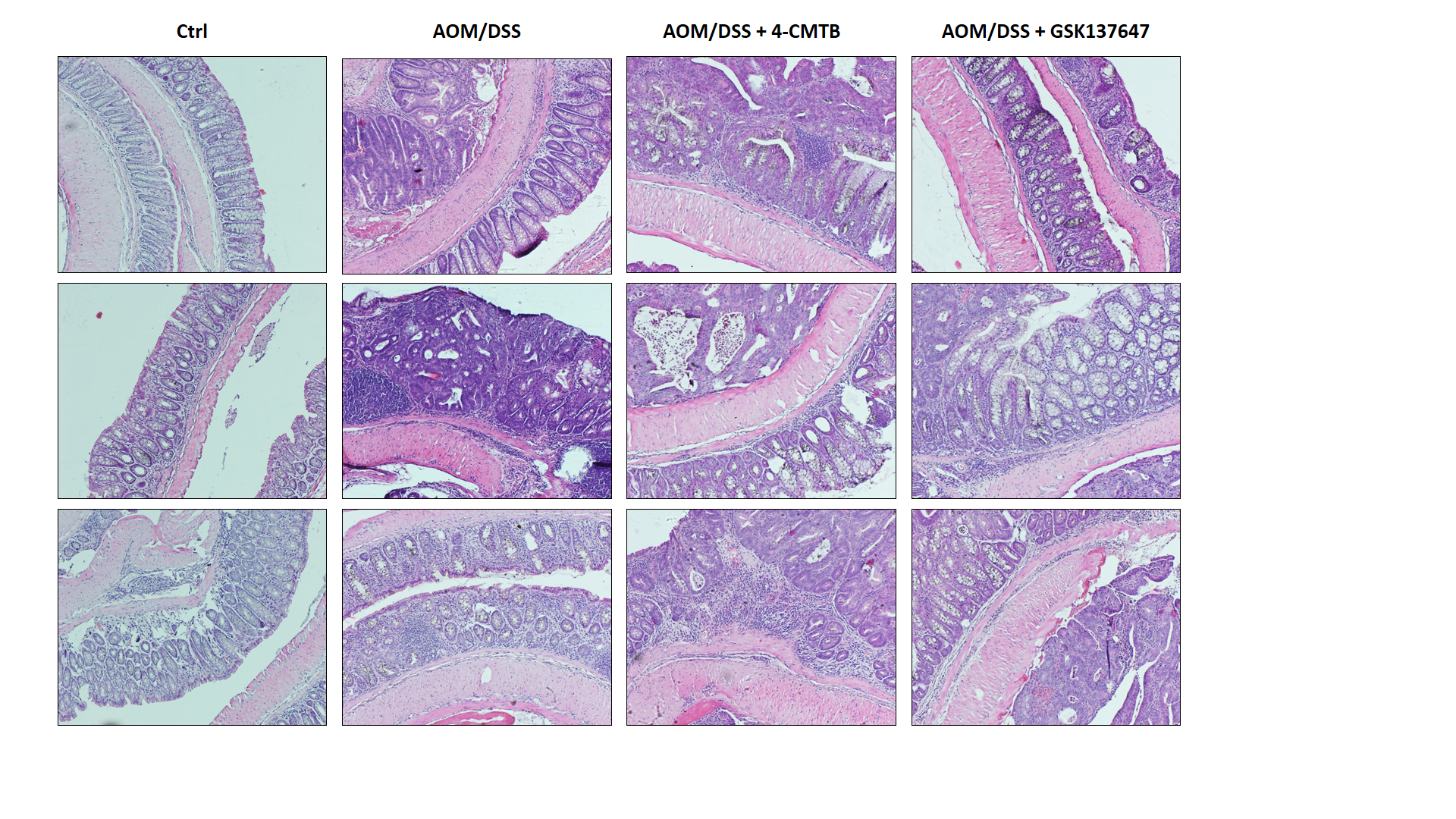


**Fig. S5** Representative histological images of the colon were collected from control, AOM/DSS, and AOM/DSS mice treated with 4-CMTB (as FFAR2 agonist) or GSK137647 (as FFAR4 agonist).

Abbreviations: AOM: azoxymethane; Ctrl: control; DSS: dextran sulfate sodium; FFAR2: free fatty acid receptor type 2; FFAR4: free fatty acid receptor type 4.


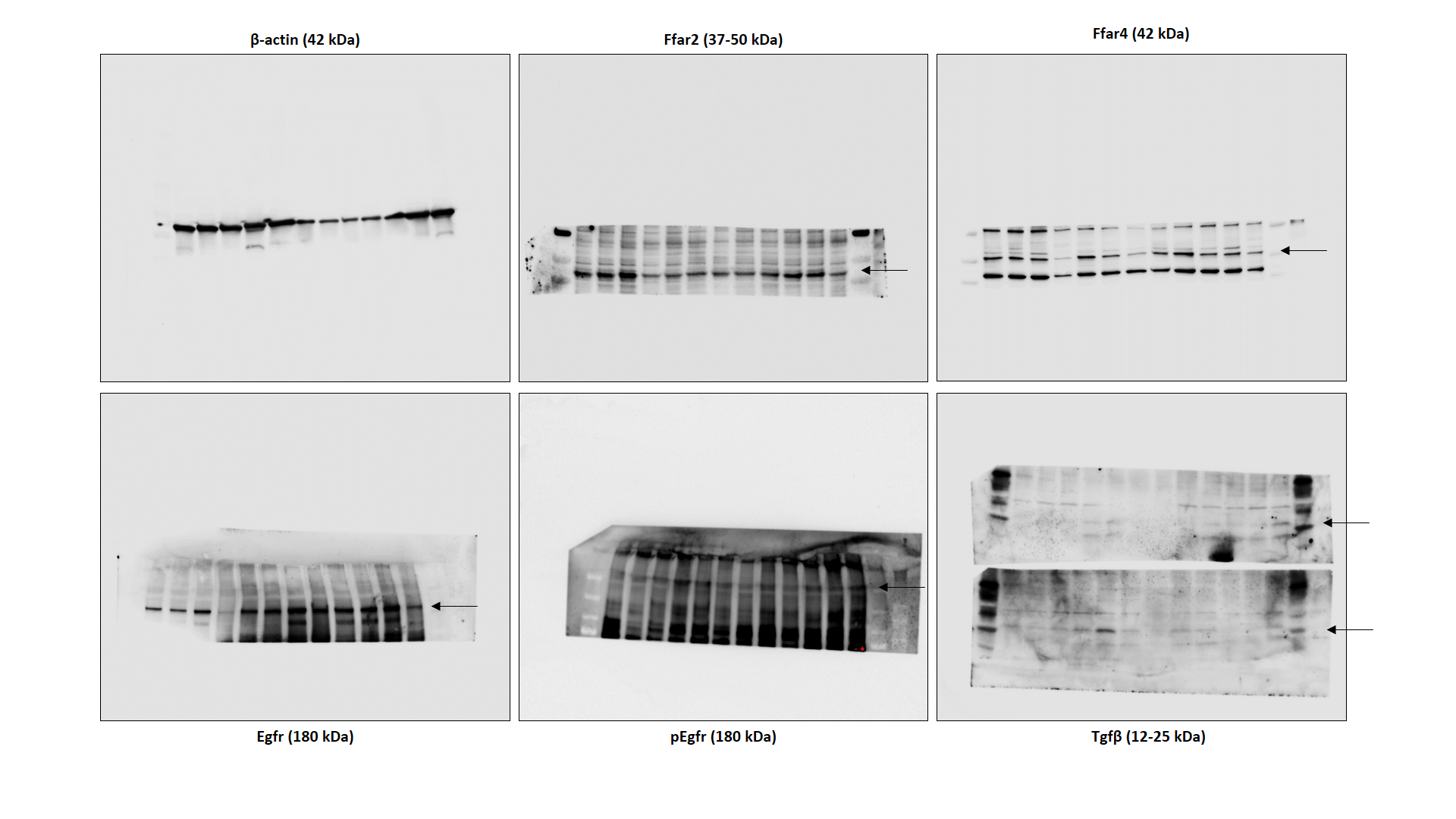


**Fig. S6** Unprocessed representative blots of β-actin, Ffar2, Ffar4, Egfr, pEgfr, and Tgfβ from *in vivo* experiment.

Abbreviations: FFAR2: free fatty acid receptor type 2; FFAR4: free fatty acid receptor type 4; Egfr: epithelial growth factor receptor; pEgfr: phosphorylated epithelial growth factor receptor; Tgfβ: transforming growth factor β.
